# Impact of red blood cell distribution width–albumin ratio on prognosis of patients with CKD

**DOI:** 10.1038/s41598-023-42986-2

**Published:** 2023-09-22

**Authors:** Hiroshi Kimura, Kenichi Tanaka, Hirotaka Saito, Tsuyoshi Iwasaki, Sakumi Kazama, Michio Shimabukuro, Koichi Asahi, Tsuyoshi Watanabe, Junichiro James Kazama

**Affiliations:** 1 Department of Nephrology and Hypertension, Fukushima Medical University, 1 Hikariga-Oka, Fukushima City, 960-1295 Japan; 2 Division of Advanced Community Based Care for Lifestyle Related Diseases, Fukushima Medical University, Fukushima City, Japan; 3 Department of Diabetes, Endocrinology and Metabolism, Fukushima Medical University, Fukushima City, Japan; 4 Division of Nephrology and Hypertension, Iwate Medical University, Morioka, Japan

**Keywords:** Cardiology, Nephrology, Risk factors

## Abstract

The red blood cell distribution width–albumin ratio (RAR) is a prognostic factor for adverse outcomes in various populations. However, whether RAR is associated with renal outcomes remains unclear. Therefore, we aimed to investigate the impact of RAR on the prognosis in patients with chronic kidney disease (CKD). We conducted a retrospective cohort study using 997 CKD patients who were enrolled in the Fukushima Cohort Study. Patients were categorized into tertiles (T1-3) according to the baseline RAR. The associations of RAR with end-stage kidney disease (ESKD) were assessed using Kaplan–Meier curves and multivariable cox regression analyses. Receiver operating characteristic (ROC) curves were performed to test whether significant differences were present between red cell distribution width (RDW) and RAR. The median age was 66, 57% were men, the median eGFR was 47.8 ml/min/1.73 m^2^, and the median value of RAR was 3.5. The higher RAR group showed an increased risk for ESKD in the Kaplan–Meier curve analysis. Compared to the lowest RAR group, higher RAR groups had a higher risk of ESKD (hazard ratio [HR] 1.37, 95% CI 0.68–2.78 and 2.92, 95% CI 1.44–5.94) for T2 and T3 groups, respectively. ROC curve analysis proved that the discriminating ability of RAR for ESKD was superior to RDW. A higher RAR value was associated with worse renal outcomes in patients with CKD. RAR could be a convenient and useful prognostic marker for renal prognosis.

## Introduction

The prevalence of chronic kidney disease (CKD) has increased worldwide in the past few decades, and 843.6 million individuals suffered from CKD in 2017^1^. CKD is a major risk factor for end-stage kidney disease (ESKD) and has emerged as one of the most prominent causes of death and morbidity in the twenty-first century^2,3^. Therefore, the early stratification of the risk of progression to ESKD is crucial in the clinical setting.

The red cell distribution width (RDW), which is known to be a marker of the heterogeneity of red blood cell volume and used for differential diagnosis of anemia^4^, has been reported to be an independent risk factor for mortality among various populations^5–8^. In terms of patients with CKD, a higher RDW was associated with an increased risk of mortality, cardiovascular events, and renal prognosis^9–11^. Generally, serum albumin is an important marker of nutritional status. Several studies have identified low albumin levels as a powerful predictor of mortality in the general population and in patients with CKD^12,13^.

Recently, the RDW to albumin ratio (RAR), which was a combined index of RDW and serum albumin, has been reported to be provided more accurate risk prediction for adverse outcomes among various populations, including heart failure, acute coronary syndrome, stroke, and sepsis^14–18^. Furthermore, recent studies have suggested that RAR may yield better results for predicting prognosis than evaluating RDW and albumin separately^16,19^.

Nevertheless, very few studies have focused on the impact of RAR on CKD progression and prognosis among patients with CKD. Therefore, we aimed to investigate the association between RAR and kidney failure requiring kidney replacement therapy over five years with annual assessments among Japanese patients with non-dialysis-dependent CKD in the Fukushima Cohort Study.

## Materials and methods

### Study population (Fukushima cohort study)

The Fukushima Cohort Study was a prospective survey and has been described in detail elsewhere^20–24^. Participants were recruited between March 2012 and July 2014, and a total of 2724 participants registered for the Fukushima Cohort study. We investigate the characteristics and outcomes (ESKD, cardiovascular events, and death) of outpatients who were under the care of specialists in nephrology or diabetology at the Fukushima Medical University Hospital.

The study was registered in the University Hospital Medical Information Network Clinical Trials Registry (UMIN-CTR) with the study ID, UMIN000040848. The study was performed in accordance with the Declaration of Helsinki. All participants provided written informed consent for participation, and the protocol was approved by the ethics committee of Fukushima Medical University (institutional review board approval numbers 1456 and 2001).

The inclusion criteria were as follows: (1) participants aged ≥ 18 years, (2) CKD patients defined as an eGFR of < 60 ml/min/1.73 m^2^ and/or proteinuria (positive dipstick results ≥ 1 +), with stable kidney function for ≥ 3 months before study entry. The eGFR was calculated using the estimation equation for Japanese patients with CKD^25^.

### Data collection and definition

Information on baseline characteristics and comorbidities (hypertension, diabetes mellitus, and dyslipidemia), as well as a history of cardiovascular disease, were obtained from the electronic medical records or the results of blood examinations at registration. RAR was calculated using the following formula: [RDW (%)/serum albumin (g/dL)]. Body mass index (BMI) was calculated as the ratio of body weight (kg) to height (m^2^). Blood pressures were measured in the sitting position using a standard sphygmomanometer or an automated device after 5 min of rest. Hypertension was defined as systolic blood pressure of ≥ 140 mmHg, diastolic blood pressure of ≥ 90 mmHg, or the use of antihypertensive medications.

Subjects with diabetes were identified by a fasting plasma glucose concentration of ≥ 126 mg/dL, glycated hemoglobin (HbA1c) value of ≥ 6.5% (National Glycohemoglobin Standardization Program), or those who used insulin or oral antidiabetic medications. Dyslipidemia was defined as triglyceride concentration ≥ 150 mg/dL, low-density lipoprotein cholesterol concentration ≥ 140 mg/dL, high-density lipoprotein cholesterol concentration < 40 mg/dL, or use of antihyperlipidemic medications.

### Exposure and outcome

The exposure of interest in this study was RAR. We categorized CKD patients into tertile groups according to the baseline RAR values described as T1, T2, and T3. The primary outcome was ESKD requiring kidney replacement therapy, and secondary outcomes were all-cause mortality and cardiovascular events, including fatal or nonfatal myocardial infarction, angina pectoris, congestive or acute heart failure, arrhythmias, cerebrovascular disorder, chronic arteriosclerosis obliterans, aortic disease, and sudden death. Information on kidney replacement therapy, deaths, and cardiovascular events were obtained from the medical record by attending physicians.

### Statistical analyses

Variables were expressed as medians with interquartile ranges or frequency (percent). Non-parametric trend tests (Jonckheere–Terpstra and Cochran–Armitage trend test) were used to evaluate the differences in baseline characteristics among the three groups^26–29^.

Kaplan–Meier curves and the log-rank test were used to compare the event-free survivals of patients by RAR levels. Then we used the Cox proportional hazards model to examine the association between the RAR and the incidence of ESKD, all-cause death, and cardiovascular events. Schoenfeld residuals were used to test the proportional hazard assumption. Additionally, receiver operating characteristic (ROC) curve analyses were performed to predict the prognostic efficiency of RAR and RDW. The following adjustments were used in these analyses: (1) Model 1, which was adjusted for age and sex; (2) Model 2, which included the above variables plus BMI, comorbidities (hypertension, diabetes, dyslipidemia), history of cardiovascular disease, and smoking; and (3) Model 3, which included the variables in Model 2 plus eGFR, proteinuria, and hemoglobin.

As secondary analyses, evaluating nonlinear associations between RAR and the incidence of ESKD, all-cause deaths, and cardiovascular events, we used Model 3-adjusted restricted cubic spline functions with four knots at the 5th, 35th, 65th, and 95th percentiles^30,31^. Also, Fine-Gray proportional hazard models were used to assess the cumulative incidence of ESKD and CV events in the presence of competing risks because of deaths before ESKD and cardiovascular events act as competing risks in CKD patients. The frequencies of missing hemoglobin, BMI, and smoking history were 0.4%, 2.7%, and 5.6%, respectively. Hence, we used multiple imputation with 20 datasets for all the analyses. All p-values < 0.05 were considered statistically significant; we used STATA MP, version 15.1 (Stata Corp, College Station, TX, USA) for all analyses.

## Results

### Participants characteristics

**Figure 1:**
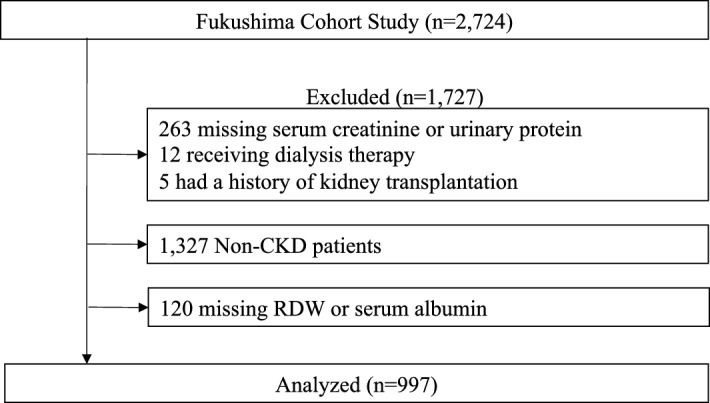
Patients flow chart. *CKD* chronic kidney disease, *RDW* red cell distribution width.

A total of 2,724 participants were registered in the Fukushima Cohort Study, and 997 patients with CKD who fulfilled the study criteria were included in this study (Fig. 1). The median age was 66 years, 57% were men, the median eGFR was 47.8 ml/min/1.73 m^2^, and the median value of RAR was 3.5. Most patients had hypertension (88%), dyslipidemia (87%), and almost half of the patients had diabetes (49%). Patients in the high RAR group were older and more likely to have diabetes. In addition, they had lower eGFR, a higher proportion of proteinuria, lower hemoglobin, and lower serum albumin than those with a low RAR group (Table 1).

**Table 1:** Baseline characteristics of 977 CKD patients in the Fukushima Cohort Study.

	Total	T1	T2	T3	P trend
RAR	3.5 (3.2–3.9)	3.1 (3.0–3.2)	3.5 (3.4–3.6)	4.2 (3.9–4.8)	< 0.001
Age, year	66 (58–75)	64 (55–73)	68 (61–76)	69 (59–77)	< 0.001
Men, %	57	54	62	54	0.946
BMI, kg/m^2^	24.1 (21.8–27.0)	24.3 (21.9–26.9)	24.3 (22.0–27.0)	23.6 (21.1–26.9)	0.147
Hypertension, %	88	85	90	89	0.09
Diabetes, %	49	37	50	61	< 0.001
Dyslipidemia, %	84	84	81	86	0.333
Cardiovascular disease history, %	14	9	17	15	0.026
Smoking history, %	52	45	59	54	0.023
eGFR, ml/min/1.73 m^2^	47.8 (34.2–56.8)	52.3 (41.8–58.9)	46.9 (33.7–56.7)	41.9 (27.1–53.3)	< 0.001
Urine protein, %	47	39	45	56	< 0.001
RDW, %	13.7 (13.1–14.4)	13.0 (12.7–13.4)	13.7 (13.3–14.2)	14.7 (14.0–15.8)	< 0.001
Hemoglobin, g/dL	12.7 (11.5–13.9)	13.6 (12.6–14.6)	12.8 (11.7–13.8)	11.7 (10.6–12.8)	< 0.001
Serum albumin, g/dL	3.9 (3.6–4.2)	4.2 (4.1–4.4)	3.9 (3.8–4.1)	3.5 (3.2–3.7)	< 0.001

Values are expressed as medians (interquartile range) or numbers and percentages as appropriate.

*RAR* red blood cell distribution width to albumin ratio, *CKD* chronic kidney disease, *BMI* body mass index, *eGFR* estimated glomerular filtration rate, *RDW* red cell distribution width.

Differences between groups were evaluated using non-parametric trend tests (Jonckheere–Terpstra, Cochran–Armitage trend test).

### RAR and the incidence of ESKD

**Figure 2:**
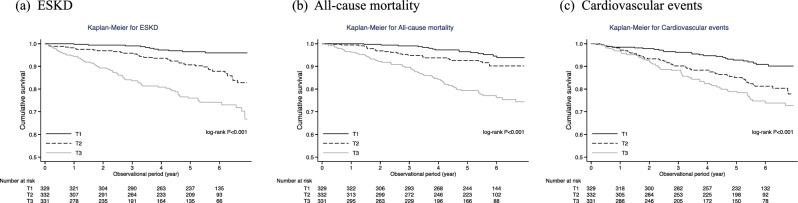
Kaplan–Meier curves for the incidence of primary and secondary outcomes categorized by the baseline RAR values. (**a**) ESKD, (**b**) All-cause mortality, (**c**) Cardiovascular events. RAR red blood cell distribution width to albumin ratio; ESKD, end-stage kidney disease.

During the median observational period of 4.6 years, 115 patients reached ESKD requiring kidney replacement therapy. Kaplan–Meier curve showed that the higher RAR group had a significantly increased risk for ESKD (Fig. 2a). Using the Cox regression model, patients with the highest RAR group (T3) showed a significantly higher risk of ESKD than those with lower RAR when we used the lowest RAR group (T1) as a reference. Although these associations were attenuated after additional adjustment, they remained significant in Model 3. The adjusted hazard ratios (HRs) were 1.37 (95% confidence interval [CI] 0.68–2.78) and 2.92 (95% CI 1.44–5.94) for T2 and T3 groups, respectively (Table 2). Similar results were observed when we used Fine-Gray proportional hazards models to assess the cumulative incidence of ESKD in the presence of competing risks (Supplemental Table).

**Table 2:** Associations between RAR and the incidence of ESKD, all-cause death, and cardiovascular events.

	Number of events	Incident rate ratio (per 100 person-year)	Unadjusted	Adjusted Hazard Ratios (95%CI)		
				Model 1	Model 2	Model 3
ESKD						
T1	11	0.63 (0.35–1.13)	Reference			
T2	36	2.25 (1.63–3.13)	3.62 (1.84–7.12)	3.71 (1.88–7.31)	3.69 (1.86–7.29)	1.37 (0.68–2.78)
T3	68	5.43 (4.28–6.89)	8.80 (4.65–16.66)	9.24 (4.88–17.53)	9.54 (4.99–18.23)	2.92 (1.44–5.94)
All-cause death						
T1	15	0.84 (0.51–1.40)	Reference			
T2	26	1.56 (1.06–2.30)	1.86 (0.99–3.52)	1.53 (0.81–2.90)	1.51 (0.79–2.88)	1.33 (0.69–2.56)
T3	62	4.34 (3.38–5.56)	5.25 (2.99–9.24)	4.58 (2.59–8.08)	4.46 (2.50–7.95)	3.38 (1.81–6.30)
Cardiovascular events						
T1	26	1.51 (1.03–2.22)	Reference			
T2	54	3.47 (2.65–4.52)	2.30 (1.44–3.67)	1.95 (1.22–3.13)	1.75 (1.09–2.82)	1.63 (1.00–2.64)
T3	62	4.68 (3.65–6.00)	3.16 (2.00–4.99)	2.90 (1.83–4.59)	2.58 (1.61–4.12)	2.27 (1.36–3.78)

Model 1: adjusted for age and sex. Model 2: adjusted Model 1 plus BMI, comorbidities (hypertension, diabetes, and dyslipidemia), history of cardiovascular disease, and smoking history. Model 3: adjusted for Model 2 plus eGFR, proteinuria, and hemoglobin.

*RAR* red blood cell distribution width to albumin ratio, *ESKD* end-stage kidney disease, *CI* confidence interval.

**Figure 3:**
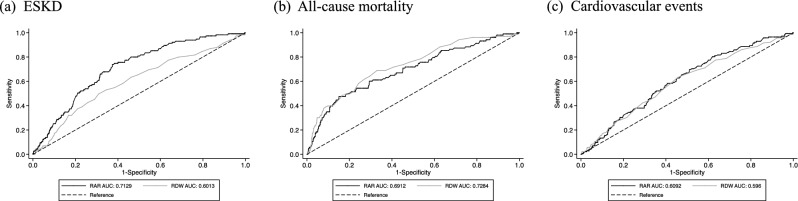
Receiver operator characteristic (ROC) curves of RAR and RDW for predicting the primary and secondary outcomes. (**a**) ESKD, (**b**) All-cause mortality, (**c**) Cardiovascular events. *RAR* red blood cell distribution width to albumin ratio, *RDW* red cell distribution width, *ESKD* end-stage kidney disease, *AUC* area under the curve.

**Figure 4:**
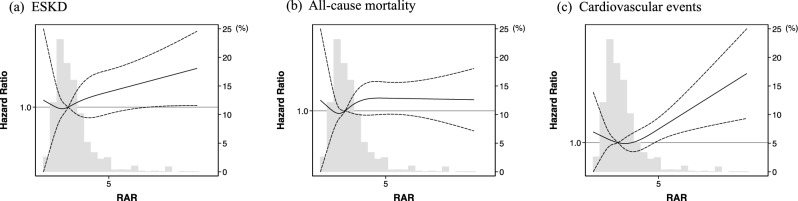
Distributions and Model 3 adjusted restricted cubic splines comparing the relationship of RAR with primary and secondary outcomes. Solid line represent adjusted hazard ratio estimates and dashed lines represent 95% CIs, respectively. (**a**) ESKD, (**b**) All-cause mortality, (**c**) Cardiovascular events. Model 3: adjusted for age, sex, BMI, comorbidities (hypertension, diabetes, and dyslipidemia), history of cardiovascular disease, smoking history, eGFR, proteinuria, and hemoglobin. *RAR* red blood cell distribution width to albumin ratio, *ESKD* end-stage kidney disease, *BMI* body mass index, *eGFR* estimated glomerular filtration rate.

The comparative analysis of ROC curves revealed that the discriminating ability of the RAR was superior to RDW (Fig. 3a). The area under the ROC curves (AUCs) for RAR and RDW were 0.713 (95%CI: 0.667–0.759) and 0.601 (95% CI: 0.544–0.658), respectively (p < 0.001). The restricted cubic spline curve showed a similar association between RAR and the incidence of ESKD (Fig. 4a).

### RAR and all-cause death, cardiovascular events

During the observational period, 103 patients died, and 142 developed cardiovascular events.

Similar to the incidence of ESKD, higher RAR groups showed significantly worse prognoses for all-cause death and cardiovascular events in Kaplan–Meier analyses (Fig. 2b,c).

Cox regression analyses showed that patients with higher RAR groups had significantly higher risks of all-cause death and cardiovascular events compared to the lowest group (T1). The adjusted hazard ratios (HRs) of T2 and T3 groups for all-cause death were 1.33 (95% CI 0.69–2.56) and 3.38 (95% CI 1.81–6.30); for cardiovascular events, were 1.63 (95% CI 1.00–2.64) and 2.27 (95% CI 1.36–3.78) (Table 2).

Although no significant difference was found between RAR and RDW by comparative analysis of ROC curves for all-cause death (p = 0.053) and cardiovascular events (p = 0.490), both showed similar AUC values (Fig. 3b,c). In addition, consistent trends were observed in all-cause mortality and cardiovascular events in restricted cubic spline analyses (Fig. 4b,c).

## Discussion

In this observational study of Japanese patients with CKD, we clarified that higher RAR was associated with an elevated risk for ESKD requiring kidney replacement therapy, all-cause death, and cardiovascular events even after adjustment for confounding variables. Moreover, the ROC curve analyses revealed that RAR had a stronger ability to predict ESKD in patients with CKD than RDW. Our findings suggest that the combination of RDW and serum albumin might be a useful marker for early risk stratification of the risk for ESKD, death, and cardiovascular events among patients with CKD.

Both RDW and serum albumin have been reported to be independent prognostic markers for various populations, including patients with CKD. Several studies reported that, high RDW levels were associated with poor renal outcomes^10,11^. Whereas the underlying mechanisms of the associations between high RDW and poor renal prognosis have still not been fully elucidated, it seems likely that these results are due to inflammation and oxidative stress. Inflammation impairs bone marrow function and iron metabolism, inhibiting erythrocyte synthesis and leading to increasing RDW levels^32^. Also, oxidative stress damages erythrocytes directly and shortens erythrocyte survival, resulting in elevated RDW levels^33,34^. Since the increased levels of inflammatory biomarkers and oxidative stress markers were associated with a faster decline in eGFR and ESKD after adjusting for known risk factors among patients with CKD, a high RDW level was likely to be associated progression of CKD. However, the inflammation and oxidative stress markers were not available in this study. Thus, we could not assess whether these markers affect RAR levels. Serum albumin is not only a nutritional marker as well an inflammation marker. Malnutrition and inflammation both reduce albumin concentration by decreasing its synthesis, and inflammation is associated with a greater fractional catabolic rate and increased transfer of albumin out of the vascular compartment^12^. Decreased serum albumin has been reported as an independent predictor of the progression of CKD, even though the slight decrease is within the normal range^13,35^.

In recent years, RAR has been reported to be a novel and useful prognostic indicator among patients with inflammatory diseases due to reflecting the pathological status of hematopoietic dysfunction and hypoalbuminemia at the same time. Furthermore, several studies have shown that RAR had more predictive power than using RDW and albumin separately^15,16^. In our study, the discriminating ability of RAR for ESKD requiring kidney replacement therapy was significantly superior to RDW. Although there was no superiority in predictive power between RAR and RDW in all-cause death and cardiovascular events, a similar area under the curve values was observed. These findings imply that RAR reflects a higher effect of low albumin on ESKD and a relatively small effect on all-cause mortality and cardiovascular events. Furthermore, the RAR can be easily obtained in laboratory tests with certain advantages of simplicity in clinical practice. Given its availability, cost-effectiveness, and high predictive value, the RAR has significance for clinicians to make quick risk stratification on patients with CKD.

The strength of this study was using a relatively large-scale population database that received specialist care for the prevention of CKD. However, we should acknowledge several limitations. First, owing to the nature of an observational study, the associations described may not reflect a cause-and-effect relationship between RAR and the progression of CKD as well as all-cause mortality and cardiovascular events. Second, RAR was assessed only at a one-time point at baseline, and changes over time were not accounted for in this study. A single measurement of the value of RAR might have led to some misclassification in this population. Third, since our study was limited to Japanese, the results cannot be generalized to all individuals with CKD.

In conclusion, we demonstrated a significant association between higher RAR and an increased risk of CKD progression, all-cause mortality, and cardiovascular events in Japanese patients with CKD. RAR can be measured at no additional cost in daily clinical practice; therefore, this variable might be convenient for identifying patients at a high risk of poor renal outcomes. However, further studies are still warranted to clarify whether aggressive interventions on RAR can improve the renal outcomes of patients with high RAR.

### Supplementary Information

Supplementary Information.

## Data Availability

The datasets generated and/or analyzed during the current study are available from the corresponding author on reasonable request.
