# Supplementary material for: Impact of red blood cell distribution width–albumin ratio on prognosis of patients with CKD
Source: Sci Rep. 2023 Sep 22;13:15774. doi: 10.1038/s41598-023-42986-2 (PMC10516924; doi:10.1038/s41598-023-42986-2)
Supplement: Supplementary file 1 — Supplementary Information. [file 41598_2023_42986_MOESM1_ESM.pdf]

Supplemental Table. Associations between RAR and the incidence of ESKD, cardiovascular events using Fine-Gray proportional hazards regression models.

			Subdistribution Hazard Ratios (95%CI)		
		**Unadjusted**	**Model 1**	**Model 2**	**Model 3**
ESKD					
	T1	Reference			
	T2	3.51 (1.79-6.91)	3.66 (1.85-7.27)	3.66 (1.84-7.25)	1.20 (0.63-2.27)
	T3	7.78 (4.12-14.69)	8.40 (4.40-16.03)	8.63 (4.58-16.25)	2.47 (1.33-4.57)
Cardiovascular events					
	T1	Reference			
	T2	2.29 (1.44-3.66)	1.95 (1.21-3.14)	1.76 (1.09-2.84)	1.65 (1.01-2.68)
	T3	2.91 (1.84-4.60)	2.64 (1.66-4.18)	2.33 (1.46-3.73)	2.09 (1.25-3.50)

Note: Model 1: adjusted for age and sex. Model 2: adjusted Model 1 plus BMI, comorbidities (hypertension, diabetes, and dyslipidemia), history of cardiovascular disease, and smoking history. Model 3: adjusted for Model 2 plus eGFR, proteinuria, and hemoglobin.

RAR, red blood cell distribution width to albumin ratio; ESKD, end-stage kidney disease; CI, confidence interval.
